# Prognostic value of mesorectal package area in patients with locally advanced rectal cancer following neoadjuvant chemoradiotherapy: A retrospective cohort study

**DOI:** 10.3389/fonc.2022.941786

**Published:** 2022-10-03

**Authors:** Bingjie Guan, Xinmin Huang, Huang Xia, Guoxian Guan, Benhua Xu

**Affiliations:** ^1^ Department of Radiation Oncology, Fujian Medical University Union Hospital, Fuzhou, China; ^2^ Department of Radiology, Fujian Medical University Union Hospital, Fuzhou, China; ^3^ Department of Colorectal Surgery, The First Affiliated Hospital of Fujian Medical University, Fuzhou, China; ^4^ Fujian Key Laboratory of Intelligent Imaging and Precision Radiotherapy for Tumors, Fujian Medical University, Fuzhou, China; ^5^ Clinical Research Center for Radiology and Radiotherapy of Fujian Province, Fuzhou, China

**Keywords:** locally advanced rectal cancer (LARC), neoadjuvant chemoradiotherapy, inflammation biomarkers, mesorectal package area, prognosis, pathology complete response

## Abstract

**Background:**

The aim of this study is to explore the most effective inflammation, magnetic resonance imaging (MRI), and nutrition markers for survival and pathology complete response (pCR) in patients with locally advanced rectal cancer (LARC).

**Methods:**

A total of 278 patients with LARC undergoing neoadjuvant chemoradiotherapy (NCRT) and radical surgery from 2016 to 2019 were included. The X-tile method was used to select the optimal cutoff points for the mesorectal package area (MPA), advanced lung cancer inflammation index (ALI), prognostic nutritional index (PNI), systemic immune-inflammation index (SII), neutrophil-to-lymphocyte ratio (NLR), platelet-to-lymphocyte ratio (PLR), and monocyte-to-lymphocyte ratio (MLR) scores. Cox regression analysis was used to identify risk factors of disease-free survival (DFS). To discover pCR risk factors, logistic regression analysis was employed. A predictive nomogram for DFS was constructed.

**Results:**

According to the least absolute shrinkage and selection operator analysis, the MPA was the only significant predictor for the DFS in patients with LARC. Kaplan-Meier (K-M) analysis demonstrated that groups with higher MPA, PNI, SII, NLR, MLR, and ALI score had improved DFS (all P < 0.05). Receiver operating characteristic (ROC) analysis revealed that the MPA and PNI could accurately predict the pCR in patients with LARC after NCRT. The MPA score and NLR score were found to be independent predictors of DFS after NCRT using Cox regression analysis. Logistical regression analysis demonstrated that the MPA score, PNI score, and pre-NCRT cN stage were all independent predictors of pCR in patients with LARC after NCRT. Recursive partitioning analysis and time-independent ROC curve analysis demonstrated that MPA score was the most important predictor of pCR and prognosis in patients with LARC after NCRT.

**Conclusions:**

MPA was identified as the most effective marker for MRI, and the prognostic value was further confirmed by time–ROC analysis. More intense adjuvant treatment could be considered for lower–MPA score patients with LARC after NCRT. Obesity in the pelvis encourages the understanding of the prognosis prediction of patients with LARC after NCRT.

## Introduction

The neoadjuvant chemoradiotherapy (NCRT) has been the standard treatment for locally advanced rectal cancer (LARC). The NCRT benefited from a higher likelihood of tumor shrinking and downstaging, enhanced tumor resectability, and better local tumor control (1–3). NCRT could contribute to pathological complete response (pCR) in 15%–27% of patients with LARC and 20%–30% near pCR in patients with LARC (4). Patients with pCR or near pCR could adopt the “watch and wait” strategy or local excision to reduce surgery-related morbidity and increase organ preservation when compared to the total mesorectal excision (TME) surgery (5–7). However, more than 30% of patients with LARC were resistant to NCRT and experiencing NCRT adverse effects (8, 9). Currently, it is still challenging to reliably estimate treatment outcomes for patients with LARC after NCRT.

The rates of obesity have risen in the recent years, and obesity contributes to a variety of chronic morbidities (10). Numerous studies have shown that obesity is associated with the occurrence and progression of colorectal cancer (11–13). However, the influence of obesity on NCRT response of LARC remains controversial (14–17). Body mass index (BMI) is the most common tool for assessing obesity, although Asians typically have normal BMI levels and abdominal obesity, which could lead to an inaccuracy evaluation. Instead of the BMI, the NCRT response may be related to the pelvic fat. Investigating pelvic fat may provide an answer to the question of whether obesity affects the NCRT responsiveness. High-resolution pelvic or rectal magnetic resonance imaging (MRI) may accurately quantify the fat in the pelvis and rectal mesorectal thickness to predict the NCRT response. Several studies found that obesity was contributing to the inflammatory response, which influenced tumor development, prognosis, and therapy response (18–20). The inflammatory indexes in the peripheral blood, NLR, MLR, PLR, and SII have been used as markers of predicting efficacy and toxicity of NCRT in patients with LARC in our previous study (21). To explore the relationship among the obesity, inflammatory response and NCRT response were important.

To address the gap in the literature, the present study aimed to explore the most effective marker of MRI measurements, systematic inflammatory, and nutrition in patients with LARC in terms of survival outcome and NCRT response.

## Patients and treatment methods

### Patients

In this study, we retrospectively analyzed 278 patients with LARC after NCRT who underwent pelvic MRI before NCRT in our hospital and radical resection between 2016 and 2019. The patient inclusion criteria and exclusion criteria were reported in our previous study (8, 22). The evaluation of the tumor staging was according to the American Joint Committee on Cancer (AJCC) (23). The TME was following the NCRT regimen, which has been described in our previous study. According to the National Comprehensive Cancer Network (NCCN) guidelines, the patients received postoperative adjuvant chemotherapy for 6 months about 1 month after surgery (24). All laboratory results and pelvic MRI images were collected within 1 week before NCRT. The last cutoff date for follow-up was 31 December 2021.

### Neoadjuvant chemoradiotherapy

Two chemotherapeutic regimens with dosages were given as follows: (1) Capox: oxaliplatin at 130 mg/m^2^ intravenously guttae, day 1; capecitabine at 825 mg/m^2^ twice daily oral, days 1–14; every 3 weeks, for two cycles during concurrent radiotherapy; another two cycles were performed during the interval from the end of radiation to surgery; (2) capecitabine alone: capecitabine at 825 mg/m^2^ twice daily oral, during the whole period of radiotherapy; another one cycle increased dosages to 1,250 mg/m^2^ was performed in 2 weeks during the waiting period.

The gross tumor volume (GTV) was calculated on the basis of clinical information, including digital rectal examination, endoscopy ultrasound, and abdominopelvic MRI. The clinical target volume (CTV) included all mesorectum, presacral soft tissue, obturator, and internal iliac lymphatic drainage regions. The planning target volume (PTV) was defined as the GTV or CTV with uniform margins of 10 mm. The neoadjuvant radiotherapy regimens consisted of three-dimensional conformal radiotherapy (3D-CRT) and intensity-modulated radiation therapy (IMRT). A dose of 50.4 Gy was delivered to PTV-GTV with 3D-CRT in 28 fractions, whereas 50 Gy was delivered with IMRT in 25 fractions. In addition, 45 Gy was delivered to PTV-CTV in 25 fractions for both types of regimens (24).

### Definitions

The pathological tumor regression grade (TRG) (23) was used as the evaluation criterion of tumor response to NCRT. No residual tumor cells in the resected specimen, including the primary site and lymph nodes, were regarded as pathological complete response (pCR). Venous blood samples were obtained within 1 week before NCRT. The following formulae were employed to determine the systematic inflammatory markers: The systemic immune-inflammation index (SII) = platelet count × neutrophil count/lymphocyte count, neutrophil-to-lymphocyte ratio (NLR) = neutrophil count/lymphocyte count, platelet-to-lymphocyte ratio (PLR) = platelet count/lymphocyte count and monocyte-to-lymphocyte ratio (MLR) = monocyte count/lymphocyte count. Advanced lung cancer inflammation index (ALI) = BMI (kg/m^2^) × albumin (g/L)/NLR, prognostic nutritional index (PNI) = serum albumin (g/L) + 5 × lymphocyte count (10^9^/L).

### Pelvic MRI measurements

**Figure 1:**
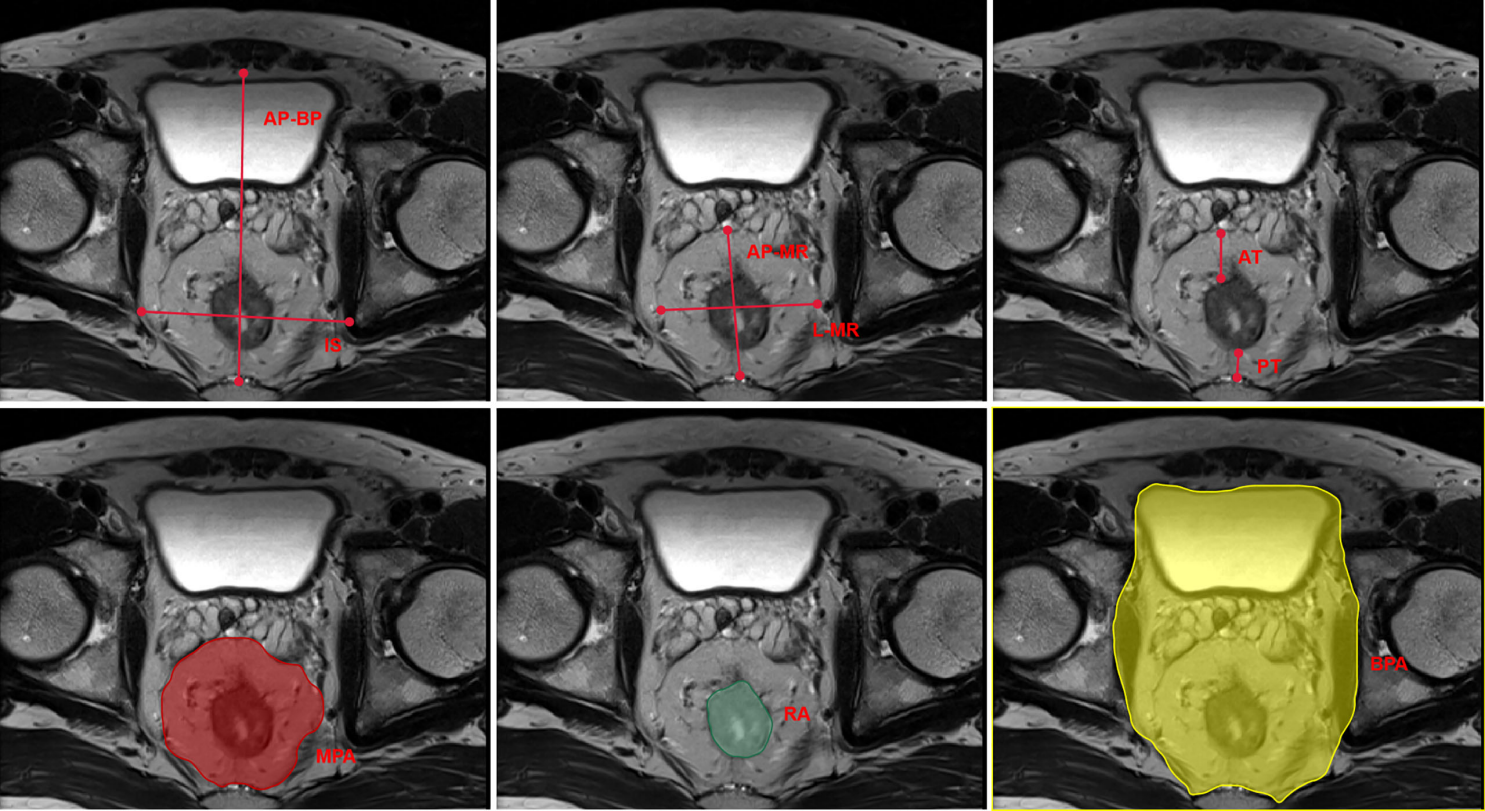
Schematic plot of the pelvic MRI measurements. MRI, magnetic resonance imaging; IS, interspinous distance; AP-BP, anterior–posterior bony pelvis span; L-MR, lateral mesorectal span; AP-MR, anterior–posterior mesorectal span; AT, anterior mesorectal thickness; PT, posterior mesorectal thickness; MPA, mesorectalpackage area; RA, rectal area; BPA, bony pelvis area.

MRI was performed using either a 1.5-T General Electric (450 W, software version 25) or a 3-T Phillips (Achieva, software version 3.2.3.5) system. Large field-of-view FT2-weighted axial images with a slice thickness of 5 mm were downloaded from the PACS system and analyzed with publicly available software (3D Slicer^©^, Version 4.11; Bethesda, MD) (25) that was supported by National Institutes of Health. The measuring procedure is shown in **Figure 1**, as described by McKechnie et al. (26).

### Statistical analysis

The Statistic Package for Social Science (SPSS, version 23.0) and R software packages version 4.0.1 were used to perform the statistical analyses. The X-tile program (http://www.tissuearray.org/rimmlab/) was used to calculate and determine the best cutoff points for the SII, NLR, PLR, MLR, ALI, and PNI counts (27). The Kaplan–Meier method and log-rank test were performed to evaluate the survival outcomes. The risk factors for overall survival (OS) and disease-free survival (DFS) were calculated by the Cox proportional hazards model. On the basis of the Cox regression model analysis, a nomogram was developed by using the R project. Time-dependent receiver operating characteristic (ROC) curves were used to evaluate the performance of the biomarkers. Least absolute shrinkage and selection operator (LASSO) Cox regression model was applied to determine the ideal coefficient for each prognostic feature and estimate the likelihood deviance (28, 29). Recursive partitioning analysis (RPA) was used to construct a decision tree that divides patients into different homogeneous risk groups by using the R project (30). Statistical significance was defined as *P* < 0.05.

## Result

### Patient characteristics

A total of 278 patients with LARC after NCRT were eligible for this analysis. There were 181 (181 of 278, 65.1%) male patients, with a mean age of 53.97 ± 10.11 years. **Tables 1**, **2** list the baseline clinicopathological characteristics of the patients.

**Table 1:** Baseline characteristics in patients with LARC after NCRT stratified by MAP.

Characteristics	MAP < 23 (n = 31)	MAP ≥ 23 (n = 247)	*P-*value
Sex (%)			1.000
Male	20 (64.5)	161 (65.2)	
Female	11 (35.5)	86 (34.8)	
Age (years)	54.35 ± 11.13	53.92 ± 10.68	0.831
ASA score (%)			0.161
1	24 (77.4)	210 (85.0.)	
2	6 (19.4)	36 (14.6)	
3	1 (3.2)	1 (0.4)	
Distance from the anal verge (cm)	6.57 ± 2.17	6.16 ± 2.36	0.357
Interval time between NCRT and surgery (weeks)	9.78 ± 1.98	9.77 ± 3.69	0.987
Pre-NCRT cT stage (%)			0.858
T2	0 (0.0)	4 (1.6)	
T3	12 (38.7)	83 (33.6)	
T4	19 (61.3)	160 (64.8)	
Pre-NCRT cN stage (%)			0.513
N0	1 (3.2)	19 (7.7)	
N+	30 (96.8)	228 (92.3)	
Pre-NCRT CEA (%)			0.443
<5.0 ng/ml	15 (48.4)	141 (57.1)	
≥5.0 ng/ml	16 (51.6)	106 (42.9)	
Pre-NCRT CA19-9 (%)			0.340
<37.0 U/ml	23 (74.2)	201 (81.4)	
≥37.0 U/ml	8 (25.8)	46 (18.6)	
Anemia (%)	6 (19.4)	38 (15.4)	0.601
Hypoproteinemia (%)	1 (3.2)	14 (5.7)	1.000

NLR, neutrophil-to-lymphocyte ratio; LARC, locally advanced rectal cancer; NCRT, neoadjuvant chemoradiotherapy; ASA, American Society of Anesthesiologists; CEA, carcinoembryonic antigen; CA19-9, carbohydrate antigen 19-9.

**Table 2:** Operative and postoperative outcomes in patients with LARC after NCRT stratified by MPA.

Characteristics	MPA < 23 (n = 31)	MPA ≥ 23 (n = 247)	*P*-value
Operative time (min)	215.32 ± 50.51	225.15 ± 66.67	0.429
Estimated blood loss (ml)	60.48 ± 29.84	80.12 ± 108.45	0.317
Surgery approach (%)			0.157
Laparoscopic	28 (90.3)	195 (78.9)	
Open	3 (9.7)	52 (21.1)	
Tumor differentiation (%)			**0.014**
Well to moderately differentiated	21 (67.7)	214(86.6)	
Poorly differentiated and others	10 (32.3)	33 (13.4)	
Postoperative hospital stay (days)	9.03 ± 5.26	8.26 ± 4.55	0.379
Postoperative complications (%)	6(19.4)	42 (17.0)	0.801
BMI			**0.034**
<18	1(3.7)	10 (4.0)	
18~24	26 (83.9)	149 (60.3)	
>24	4 (12.9)	88 (35.6)	
Organ preservation (%)	30 (96.8)	230 (93.1)	0.703
Tumor size (cm)	2.53 ± 1.01	2.61 ± 1.26	0.767
Pathological T stage (%)			**0.001**
T0	1 (3.2)	70 (28.3)	
T1	0 (0.0)	16 (6.5)	
T2	11 (35.5)	60 (24.3)	
T3	16 (51.6)	97 (39.3)	
T4	3 (9.7)	4 (1.6)	
Pathological N stage (%)			0.122
N0	19 (61.3)	185 (74.9)	
N1	8 (25.8)	50 (20.2)	
N2	4 (12.9)	12 (4.9)	
Pathological M stage (%)			**0.011**
M0	27 (87.1)	242 (98.0)	
M1	4 (12.9)	5 (2.0)	
TRG (%)			**0.022**
0	1 (3.2)	67 (27.1)	
1	11 (35.5)	80 (32.4)	
2	17 (54.8)	85 (34.4)	
3	2 (6.5)	15 (6.1)	
pCR rates (%)	1 (3.2)	67 (27.1)	**0.002**
Nerval invasion (%)	5 (16.1)	18 (7.3)	0.155
Vascular invasion (%)	2 (6.5)	10 (4.0)	0.630
NLR score	4.71 ± 7.87	2.65 ± 2.10	**0.001**
SII score	1276.87 ± 2761.05	691.51 ± 693.10	**0.007**
MLR score	0.34 ± 0.21	0.27± 0.15	**0.022**
PLR score	161.34 ± 82.56	149.79 ± 97.47	0.528
PNI score	48.41 ± 4.20	49.75 ± 5.13	0.164
ALI score	42.97 ± 34.38	49.51 ± 26.42	0.212

LARC, locally advanced rectal cancer; NCRT, neoadjuvant chemoradiotherapy; NLR, neutrophil-to-lymphocyte ratio; TRG, tumor regression grade; pCR, pathological complete respons; SII, systemic immune-inflammation index; MLR, monocyte-to-lymphocyte ratio; PLR, platelet-to-lymphocyte ratio. P<0.05 was statistically significant in bold.

### The LASSO analysis

**Figure 2:**
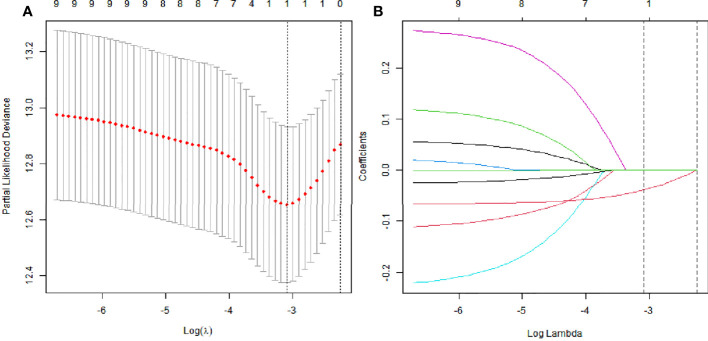
The least absolute shrinkage and selection operator (LASSO) analysis and risk score system were constructed. **(A)** The area under the ROC curve (AUC) was estimated with a cross-validation technique, and the largest lambda value was chosen when the cross-validation error was within one standard error of the minimum. **(B)** LASSO coefficient profiles of the eight factors.

**Figure 3:**
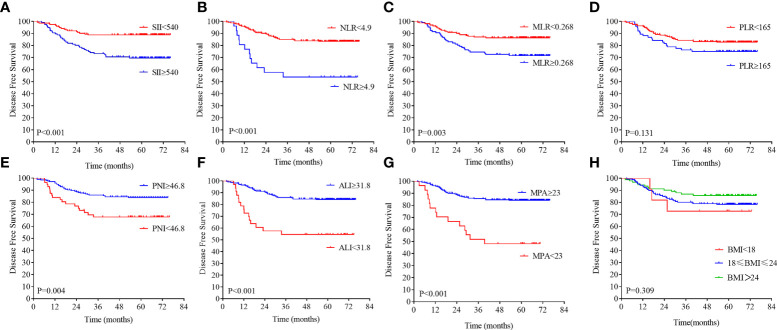
Kaplan–Meier analysis of the NLR, SII, PLR, MLR, PNI, ALI, MPA, and BMI level. The disease-free survival for the optimal cutoff point of the SII **(A)**, NLR **(B)**, MLR **(C)**, PLR **(D)**, PNI **(E)**, ALI **(F)**, MPA **(G)**, and BMI level **(H)**.

**Figure 4:**
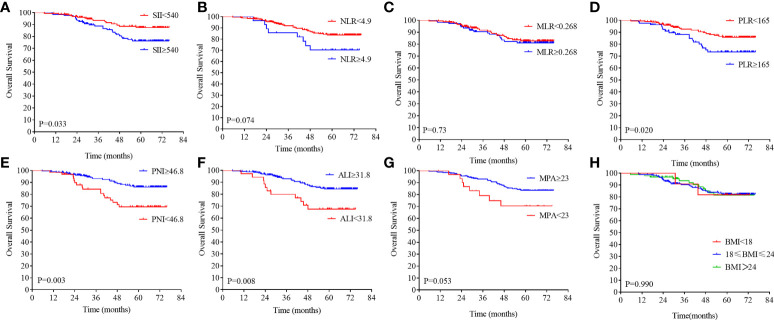
Kaplan–Meier analysis of the NLR, SII, PLR, MLR, PNI, ALI, MPA, and BMI level. The overall survival for the optimal cutoff point of the SII **(A)**, NLR **(B)**. MLR **(C)**, PLR **(D)**, PNI **(E)**, ALI **(F)**, MPA **(G)**, BMI **(H)**.

LASSO analysis was used to explore significant predictors in MRI measurement markers for DFS in the patients with LARC after NCRT. The result demonstrated that the mesorectal package area (MPA) was the only factor that mattered (**Figures 2A, B**). Furthermore, the X-tile plot was employed to select the optimal cutoff point for the MPA, with the outcome revealing the cutoff values of 23 for MPA (**Supplementary Figure 1**). In addition, the best optimal cut-off point for the MPA was enrolled in the next analysis. The result demonstrated that a high value of the MPA had better prognosis in the patients with LARC (DFS, P < 0.01, **Figure 3G**; OS, P = 0.05, **Figure 4G**).

### Association of inflammation and nutrition biomarkers with survival

On the basis of the DFS, the X-tile plots were constructed and identified 540, 4.9, 0.268, 165, 46.8, and 31.8 as the cutoff values for SII, NLR, MLR, PLR, PNI, and ALI, respectively. Then, we divided the entire cohort into low and high subgroups. As shown in **Figure 3**, higher PNI and ALI scores were associated with better DFS in patients with LARC. DFS rates were significantly greater in the high PNI and ALI score group at 3 years, at 85.9% and 85.9%, respectively, than in the low PNI and ALI score group, at 67.7% and 54.5%, respectively (all P = 0.01; **Figures 3E, F**). Moreover, a high score of the SII, NLR, PLR, and MLR was correlated with worse DFS in patients with LARC compared with the low SII, NLR, PLR, and MLR score group, as shown in **Figures 3A–D**. The DFS rates at 3 years for the high SII, NLR, PLR, and MLR group were 73.2%, 53.8%, 76.3%, and 74.5%, respectively, significantly lower than 88.8%, 77.6%, 85.1%, and 87.1% in the low SII, NLR, PLR, and MLR groups, respectively (P < 0.01, P < 0.01, P = 0.13, and P < 0.01, respectively). Noticeably, the high PNI and ALI score groups had better OS compared with the low score group, as shown in **Figures 4E, F** (all P < 0.01). In addition, low SII and PLR score group had significantly better OS than the high score group (SII: P = 0.03, **Figure 4A**; PLR: P = 0.02, **Figure 4D**). There was no statistical difference between the low NLR and MLR score groups and the high NLR and MLR score groups (NLR: P = 0.07, **Figure 4B**; MLR: P = 0.73, **Figure 4C**). Moreover, the BMI level was not associated with the DFS and OS in the patients with LARC (DFS: P = 0.31, **Figure 3H**; OS: P = 0.99, **Figure 4H**).

### Association of biomarkers with pCR

**Figure 5:**
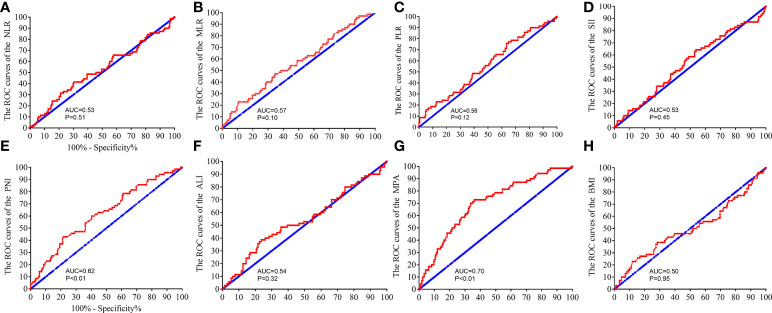
Receiver operating characteristic (ROC) analysis to evaluate the predictive efficiency of the NLR, SII, PLR, MLR, PNI, ALI, MPA, and BMI level in patients with LARC for NCRT response. The ROC analysis for NLR **(A)**, MLR **(B)**, PLR **(C)**, SII **(D)**, PNI **(E)**, ALI **(F)**, MPA **(G)**, and BMI level **(H)**.

The correlations between pathological complete response (pCR) and MRI, inflammatory and nutritional biomarkers were further explored. The ROC analysis was performed to verify the predicting ability of the biomarkers. The MPA and PNI scores had powerful ability to predict the pCR in the patients with LARC [PNI: area under the ROC curve (AUC) = 0.62, P < 0.01, **Figure 5E**; MPA: AUC = 0.70, P < 0.01, **Figure 5G**]. However, the other biomarkers could not predict the pCR in the patients with LARC after NCRT (NLR: AUC = 0.53, P = 0.51, **Figure 5A**; MLR: AUC = 0.57, P = 0.10, **Figure 5B**; PLR: AUC = 0.56, P = 0.12, **Figure 5C**; SII: AUC = 0.53, P = 0.45, **Figure 5D**; ALI: AUC = 0.54, P = 0.32, **Figure 5F**; BMI: AUC = 0.50, P = 0.94, **Figure 5H**).

### Association of MPA with clinicopathological characteristics

On the basis of the optimal cutoff value, these patients were dichotomized into the low-MPA group (n = 31, 11.1%) and the high-ALI group (n = 247, 88.9%). No significant differences were found between the groups regarding baseline characteristics, such as sex, age, American Society of Anaesthesiologists (ASA) score, preoperative carcinoembryonic antigen (CEA) level, preoperative CA19-9 level, distance from the anal verge, interval time between NCRT and surgery, pre-NCRT cT stage, pre-NCRT cN stage, hypoproteinemia, estimated blood loss, operative time, postoperative hospital stay, organ preservation, tumor size, BMI level, and anemia (all P > 0.05, **Tables 1**, **2**). As expected, a lower MAP level was associated with poorly tumor differentiation, higher pathology TNM stage, higher AJCC TRG stage, higher NLR score, higher MLR score, and higher SII score (all P < 0.05).

### Prognostic value of the biomarkers

To explore the prognostic impact of the biomarkers on DFS in patients with LARC, we performed a Cox regression model analysis. On univariate analysis, pathological T stage (P < 0.001), pathological N stage (P < 0.001), AJCC TRG grade (P = 0.001), pre-NCRT CEA level (P = 0.017), MPA score (P < 0.001), NLR score (P < 0.001), MLR score (P < 0.001), PNI score (P = 0.005), ALI score (P = 0.001), nerval invasion (P = 0.006), and tumor differentiation (P = 0.002) were independently associated with DFS in patients with LARC after NCRT and TME (**Table 3**). Results from the multivariate Cox regression model demonstrated that MPA score [hazard ratio (HR) = 0.954; 95% confidence interval (CI), 0.921–0.988; P = 0.009] and NLR level (HR = 1.058; 95% CI, 1.004–1.115; P = 0.034) were independent predictors of DFS after NCRT (**Table 3**).

**Table 3:** Cox regression analysis of predictive factors for disease-free survival in patients with LARC after NCRT (n = 278).

Variables	Univariate analysis			Multivariate analysis		
	HR	95% CI	*P*-value	HR	95% CI	*P*-value
Sex, male/female	1.049	0.622–1.769	0.858			
Age	0.986	0.964–1.009	0.232			
ASA	0.978	0.508–1.883	0.948			
Postoperative hospital stay	1.002	0.949–1.057	0.947			
Distance from the anal verge	0.999	0.898–1.112	0.989			
Tumor size	0.843	0.679–1.048	0.125			
Pathological T stage	1.734	1.332–2.258	**<0.001**	1.380	0.945–2.015	0.095
Pathological N stage	2.023	1.426–2.870	**<0.001**	1.153	0.751–1.771	0.515
AJCC TRG grade	1.675	1.246–2.253	**0.001**	1.020	0.633–1.645	0.935
Interval time between NCRT and surgery	0.908	0.778–1.061	0.224			
Pre-NCRT cT stage	1.180	0.722–1.931	0.509			
Pre-NCRT cN stage	0.869	0.668–1.131	0.295			
Operative time	1.003	0.999–1.007	0.113			
Estimated blood loss	0.999	0.996–1.002	0.553			
Pre-NCRT CEA level	1.860	1.119–3.089	**0.017**	1.506	0.883–2.569	0.133
Pre-NCRT CA19-9 level	1.458	0.814–2.612	0.205			
Anemia	1.369	0.728–2.574	0.329			
Hypoproteinemia	1.660	0.665–4.144	0.278			
MPA score	0.926	0.896–0.957	**<0.001**	0.954	0.921–0.988	**0.009**
BMI	0.928	0.852–1.010	0.082			
NLR score	1.090	1.053–1.128	**<0.001**	1.058	1.004–1.115	**0.034**
SII score	1.000	1.000–1.001	0.078			
MLR score	9.954	3.015–32.856	**<0.001**	1.157	0.222–6.029	0.862
PLR score	1.001	1.000–1.003	0.099			
PNI score	0.930	0.884–0.979	**0.005**	0.990	0.975–1.004	0.156
ALI score	0.980	0.969–0.992	**0.001**	1.013	0.949–1.080	0.707
Organ preservation	1.353	0.424–4.319	0.609			
Postoperative complications	1.661	0.972–2.975	0.088			
Nerval invasion	2.594	1.316–5.113	**0.006**	1.616	0.778–3.358	0.198
Vascular invasion	1.692	0.614–4.663	0.310			
Tumor differentiation	2.374	1.356–4.157	**0.002**	1.550	0.860–2.791	0.145

LARC, locally advanced rectal cancer; NCRT, neoadjuvant chemoradiotherapy; HR, hazard ratio; CI, confidential interval; ASA, American Society of Anesthesiologists; AJCC, American Joint Committee on Cancer; CEA, carcinoembryonic antigen; CA19-9, carbohydrate antigen 19-9; NLR, neutrophil-to-lymphocyte ratio; SII, systemic immune-inflammation index; MLR, monocyte-to-lymphocyte ratio; PLR, platelet-to-lymphocyte ratio. P<0.05 was statistically significant in bold.

### Univariate and multivariate analysis of pCR

**Figure 6:**
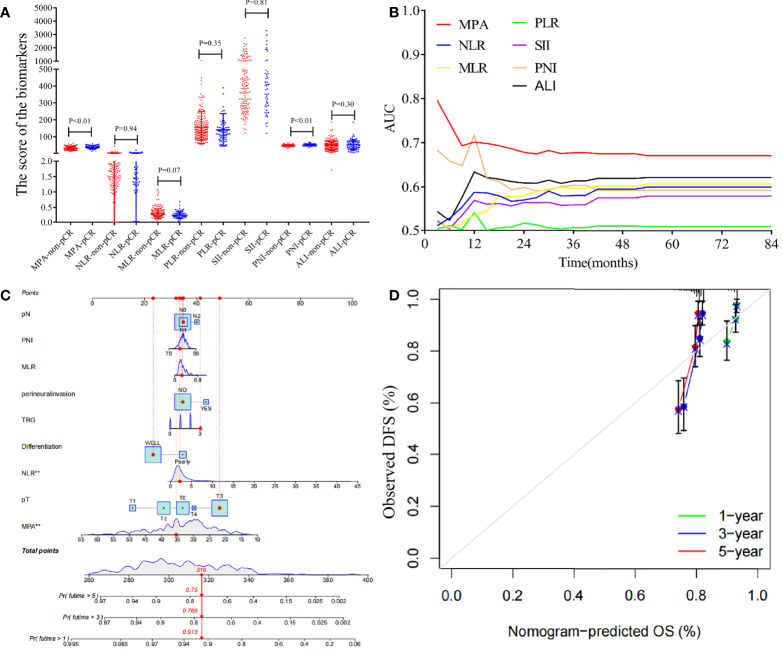
Analysis the biomarkers in the patients with LARC and construction a nomogram model for the disease-free survival. **(A)** The biomarkers value in the pCR group and non-pCR group (MPA: pCR, 37.98 ± 9.02, vs. non-pCR, 31.86 ± 8.62, P < 0.01; NLR: pCR, 2.89 ± 2.94, vs. non-pCR, 2.88 ± 3.45, P = 0.94; SII: pCR, 783.12 ± 1,090.10, vs. non-pCR, 748.26 ± 1,150.48, P = 0.81; PLR: pCR, 141.4 ± 98.22, vs. non-pCR, 154.22 ± 95.11, P = 0.35; MLR: pCR, 0.25 ± 0.12, vs. non-pCR, 0.29 ± 0.17, P = 0.07; PNI: pCR, 51.31 ± 5.33, vs. non-pCR, 49.05 ± 4.84, P < 0.01; ALI: pCR, 52.46 ± 30.4, vs. non-pCR, 47.59 ± 26.36, P = 0.30). **(B)** Time-dependent AUC curves of the NLR, SII, PLR, MLR, PNI, ALI, and MPA, for the prediction of disease-free survival. **(C)** Nomogram developed for prediction of disease-free survival. **(D)** Calibration curves for 1-, 3-, and 5-year DFS for the nomogram in patients with LARC after NCRT.

The score of the biomarkers in the pCR group and non-pCR group was compared by the T-test, as shown in **Figure 6A**. The result demonstrated that the MPA and PNI scores were significantly higher in the pCR group compared with that in the non-pCR group (MPA: pCR, 37.98 ± 9.02, vs. non-pCR, 31.86 ± 8.62, P < 0.01; PNI: pCR, 51.31 ± 5.33, vs. non-pCR, 49.05 ± 4.84, P < 0.01). However, another biomarkers score was no significant association with the pCR or non-pCR group (NLR: pCR, 2.89 ± 2.94, vs. non-pCR, 2.88 ± 3.45, P = 0.94; SII: pCR, 783.12 ± 1,090.10, vs. non-pCR, 748.26 ± 1,150.48, P = 0.81; PLR: pCR, 141.4 ± 98.22, vs. non-pCR, 154.22 ± 95.11, P = 0.35; MLR: pCR, 0.25 ± 0.12, vs. non-pCR, 0.29 ± 0.17, P = 0.07; ALI: pCR, 52.46 ± 30.4, vs. non-pCR, 47.59 ± 26.36, P = 0.30). To explore the impact of the biomarkers on pCR in patients with LARC, we performed a logistical regression model analysis. On univariate analysis, pre-NCRT cT stage (P = 0.023), MPA score (P < 0.001), pre-NCRT cN stage (P = 0.006), and PNI score (P = 0.002) were independently associated with pCR in patients with LARC (**Table 4**). The multivariate logistical regression model demonstrated that MPA score (OR = 0.926; 95% CI, 0.895–0.958; P < 0.001), PNI score (OR = 0.925; 95% CI, 0.871–0.983; P = 0.011), and pre-NCRT cN stage (OR = 1.634; 95% CI, 1.177–2.269; P = 0.034) were independent predictors of pCR after NCRT (**Table 4**).

**Table 4:** Logistic regression analysis of predictive factors for pCR in patients with LARC after NCRT (n = 278).

Variables	Univariate analysis			Multivariate analysis		
	OR	95% CI	*P*-value	OR	95% CI	*P*-value
Sex, male/female	0687	0.393–0.198	0.186			
Age	0.991	0.966–1.017	0.508			
ASA	0.950	0.478–1.886	0.883			
Distance from the anal verge	0.976	0.870–1.096	0.682			
Tumor size	0.964	0.775–1.199	0.741			
MPA score	0.926	0.897–0.956	<**0.001**	0.926	0.895–0.958	<**0.001**
BMI	1.001	0.917–1.093	0.978			
Interval time between NCRT and surgery	0.996	0.925–1.073	0.924			
Pre-NCRT cT stage	1.777	1.082–2.919	**0.023**	1.587	0.920–2.735	0.097
Pre-NCRT cN stage	1.524	1.129–2.057	**0.006**	1.634	1.177–2.269	**0.003**
Postoperative hospital stay	0.995	0.939–1.054	0.856			
Pre-NCRT CEA level	1.856	1.052–3.276	**0.033**	1.550	0.835–2.878	0.165
Pre-NCRT CA19-9 level	1.398	0.676–2.889	0.366			
Anemia	1.622	0.715–3.680	0.247			
Hypoproteinemia	1.367	0.374–4.993	0.636			
NLR score	0.997	0.920–1.080	0.943			
SII score	1.000	1.000–1.001	0.809			
MLR score	6.905	0.844–56.527	0.072			
PLR score	1.002	0.998–1.005	0.354			
PNI score	0.913	0.863–0.967	**0.002**	0.925	0.871–0.983	**0.011**
ALI score	0.995	0.985–1.005	0.301			
Tumor differentiation	2.308	0.930–5.729	0.071			

CI, confidential interval; ASA, American Society of Anesthesiologists; AJCC, American Joint Committee on Cancer; CEA, carcinoembryonic antigen; CA19-9, carbohydrate antigen 19-9; NLR, neutrophil-to-lymphocyte ratio; SII, systemic immune-inflammation index; MLR, monocyte-to-lymphocyte ratio; PLR, platelet-to-lymphocyte ratio. P<0.05 was statistically significant in bold.

### Predictive models for DFS

The time-dependent ROC curves of the biomarkers showed that all the AUCs were relatively stable after surgery during the observation period. However, the AUC of the MPA tended to be higher than the other biomarkers at all times tested (**Figure 6B**). Based on the above important factors of logistics regression, a nomogram was constructed to predict DFS in LARC patients (**Figure 6C**). The 3-year DFS predictive probabilities were obtained by drawing a straight line after summing up the score of each variable (**Figures 6D**). Patients with a higher total score tended to have lower DFS rate.

### RPA to identify high-risk and low-risk groups of pCR

**Figure 7:**
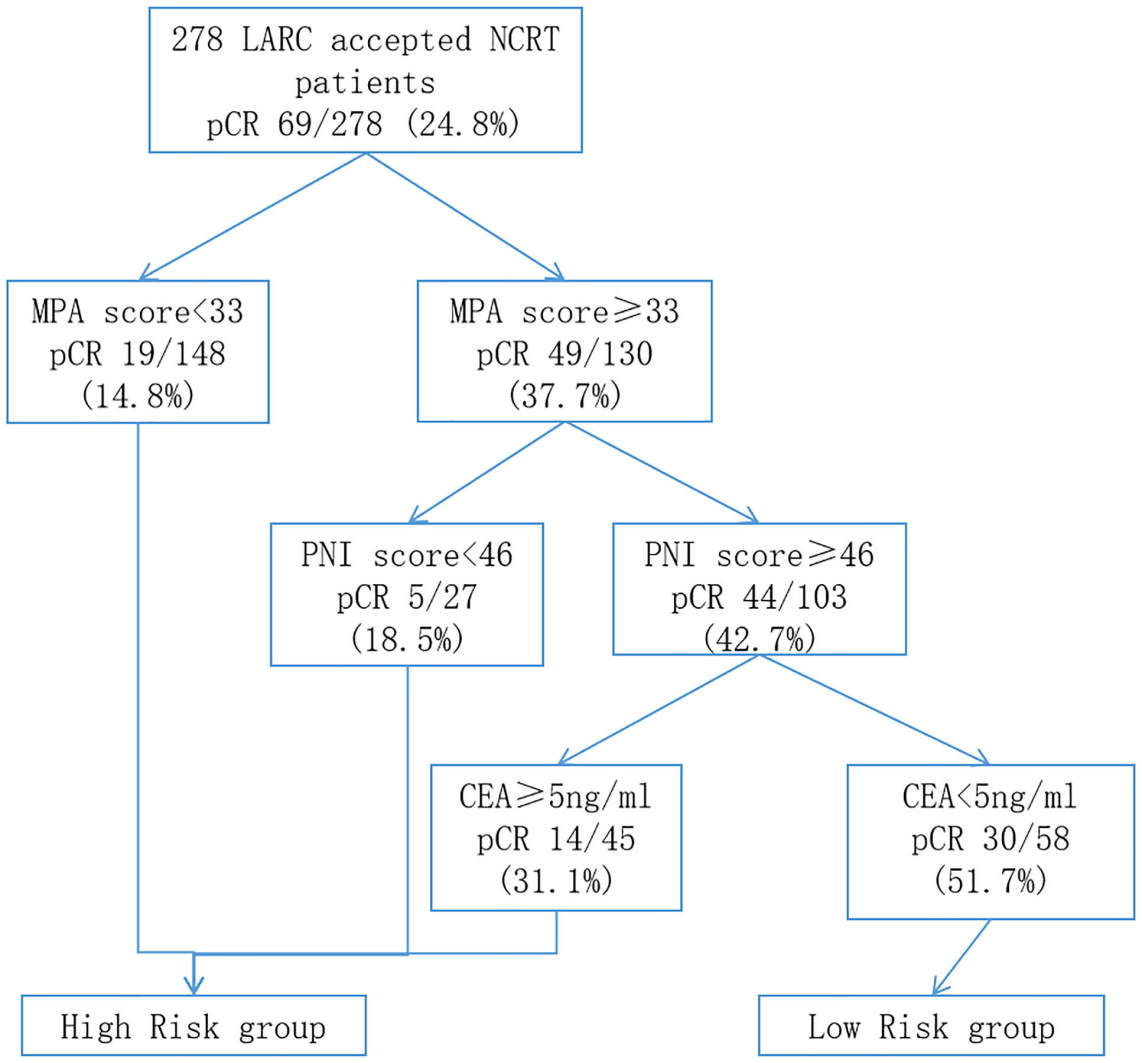
Classification tree identifying the groups at high- and low-risk for pCR of patients with LARC after NCRT. pCR, pathology complete response; LARC, locally advanced rectal cancer; NCRT, neo-chemoradiotherapy.

On the basis of the results of the multivariate logistical regression analysis, RPA was performed, and patients with LARC after NCRT were divided into different pCR rate groups (**Figure 7**). The independent risk factors included in the RPA were MPA score, PNI score, and pre-NCRT CEA level. On the basis of the above three factors, the patients were divided into four groups. The model showed that the MPA score was the most important factor affecting pCR. When the MPA score is under 33, the pCR rate remains at 14.8%. In contrast, the MPA score of more than 33, the pCR rate was 37.7%. Moreover, we found the similar result that the PNI score is more than 46, resulting in the pCR rates of 42.7%. In addition, on the basis of the pre-NCRT CEA level, the patients with LARC were divided into two groups. Finally, the pCR rates in the low-risk group patients were 51.7%, whereas the pCR rates in the high-risk group patients were 17.7%, and the difference was significant (p < 0.001).

## Discussion

The occurrence rate of obesity is increasing in the worldwide especially in China (31, 32). Recently, many studies reported that obesity contributed to developing multiple cancers and a worse prognosis (18, 33). Controversially, several studies revealed that patients with obesity had greater NCRT response when they had rectal cancer (34, 35). To assess obesity, a number of measurements are available, including BMI, waistline, and visceral adipose tissue. The above measurements are aimed to determining body fat. However, the NCRT range of irradiation for patients with LARC was limited to the pelvic and rectal. Thus, whether the body fat can instead of the pelvic and rectal fat is yet uncertain. The current study aims to evaluate the pelvic and rectal fat to predict NCRT response and prognosis in patients with LARC.

There was not standard for correctly assessing pelvic and rectal fat until now. McKechnie et al. (26) reported a better way to evaluate pelvic and rectal fat using MRI to measure the area of the fat in the pelvic. Moreover, radiomics shows multiple advantages in evaluating NCRT response in LARC (36–38). According to the NCCN and ESMO guidelines, high-resolution pelvic or rectal MRI could be an efficient routine imaging tool for evaluating clinical tumor stage and NCRT response. In the present study, we analyzed the pelvic parameters combined with radiomics, and on the basis of the LASSO analysis, the MPA was selected as the effective biomarker to predict the prognosis.

MPA includes the mesorectal and rectal thicknesses, and the rectal thickness is usually steady, so the mesorectal thickness determined the MPA score. Posterior mesorectal thickness is an important factor influencing operative complexity in rectal surgery (26, 39). Patients with the hypertrophy mesorectal might obscure anatomic dissection planes or limit access to the pelvis, potentially increasing the technical challenge of rectal surgery particularly for the patients with LARC after NCRT (40). The MPA score is connected with the BMI level and may represent pelvic obesity in the current study. Investigating the impact of the MPA score in patients with LARC may shed light on the involvement of obesity in the pelvis. On the basis of the MPA high- and low score groups, we found that the high MPA score group was associated with a low pathology TNM stage and high rates of the pCR. However, the BMI level could not distinguish the above results well. The result revealed that, rather than BMI, the pelvic obesity may contribute to the NCRT response. Moreover, the Cox regression and logistical regression also identified that the MPA score was crucial in predicting NCRT response and prognosis in patients with LARC.

Several studies found that nutrition and inflammation are related to tumor development and progression (41, 42). Obesity and albumin have been recognized as essential parameters for evaluating the nutritional status of patients with cancer (43). At present, obesity is associated with inflammatory response and affects the efficiency and toxicity of chemotherapy and radiotherapy in patients with cancer (44–46). The inflammatory indexes in the peripheral blood, NLR, MLR, PLR, and SII have been used as markers of predicting efficacy and toxicity of NCRT in patients with LARC in our previous study (21). Furthermore, mounting evidence suggested that obesity was contributing to the inflammatory response, which influenced tumor development, prognosis, and therapy response (18–20). As a result, we hypothesize that the pelvic fat increases the inflammatory response to affect the NCRT response. To further explore the relationship between the pelvic obesity and inflammatory response, we analyze the relationship between the MPA score and NLR, PLR, MLR, SII, PNI, and ALI. The result showed that the MPA score was associated with the NLR, PLR, and MLR score. There were more pieces of evidence that pelvic fat was related with the inflammatory response. PNI is a novel index to reflect the nutritional and inflammatory status of patients, and its clinical efficacy as a predictive factor in different malignancies has been established (47, 48). In the present study, we found that both the PNI and MPA scores were effective at predicting NCRT response in patients with LARC. However, only MPA was associated with NCRT response in logistic regression analysis. This could imply that pelvic fat modulates inflammatory response to elicit NCRT response.

To predict the pCR rates of the patients with LARC after NCRT, the RPA was performed to classify patients with LARC into different risk groups. RPA was a useful statistical method for predicting patient risk in a number of cancers, including colorectal cancer, nasopharyngeal cancer, cervical cancer, and breast cancer, which could assist clinicians to determine the best medication regimen (30, 47–49). However, few studies used the RPA to forecast the NCRT response in patients with LARC. In the present study, the MPA score, PNI score, and pre-NCRT CEA level play an important role in dividing the patients into the different risk groups. Among the affecting criteria, the MPA score has the most significant influence. On the basis of the MPA score, we distinguished over half of the patients with LARC in the first step and then selected 20% of patients as the low-risk patients, who may accept a greater pCR rates than the high-risk group, based on the PNI score and pre-NCRT CEA level. The results mentioned provide fresh treatment options for patients with LARC after NCRT.

Several limitations warrant discussion. First, the present study was subjected to potential selection bias due to the retrospective design. In addition, limitations in statistical methods resulted in imbalanced grouping of the groups. Second, peripheral blood cell analysis results might be affected by factors, such as blood circulation capacity, infection, and nutritional status. Third, the impact of gene profiling and tumor microenvironment inflammation was not assessed, owing to the lack of complete medical records. Despite these limitations, we believe that this study adds to the understanding of the impact of pelvic obesity on the oncological outcomes in patients with LARC after NCRT.

In conclusion, a higher MPA score was associated with poorer DFS and OS in patients with LARC after NCRT. In addition, MPA score was identified to be the most reliable marker, and the prognostic value was further confirmed by time–ROC analysis. Finally, an RPA was constructed to predict the DFS outcomes. Patients in the high-risk group who have LARC after NCRT may benefit from more intensive adjuvant therapy. Larger-scale prospective clinical trials are warranted to support the above findings.

## Data availability statement

The raw data supporting the conclusions of this article will be made available by the authors, without undue reservation.

## Ethics statement

Written informed consent was obtained from the individual(s) for the publication of any potentially identifiable images or data included in this article.

## Author contributions

All authors made a significant contribution to the present study, whether that is in the conception, study design, execution, acquisition of data, analysis and interpretation, or in all these areas; took part in drafting, revising, or critically reviewing the article; gave final approval of the version to be published; have agreed on the journal to which the article has been submitted; and agree to be accountable for all aspects of the work.

## Funding

This study was sponsored by Fujian provincial health technology project (2021CXA011 and 2021CXB009); National Foundation of China (No. 82172800); Science Foundation of the Fujian Province (No. 2019J01161); Special Financial Foundation of Fujian Provincial (No. 2020B019); Joint Funds for the innovation of science and Technology, Fujian Province (2020Y9125); and the Talent programs granted from The First Affiliated Hospital of Fujian Medical University (YJRC3600).

## Acknowledgments

The authors thank all the staff in the Fujian Medical University Union Hospital, Fuzhou, Fujian Province, People’s Republic of China.

## Conflict of interest

The authors declare that the research was conducted in the absence of any commercial or financial relationships that could be construed as a potential conflict of interest.

## Publisher’s note

All claims expressed in this article are solely those of the authors and do not necessarily represent those of their affiliated organizations, or those of the publisher, the editors and the reviewers. Any product that may be evaluated in this article, or claim that may be made by its manufacturer, is not guaranteed or endorsed by the publisher.
