# Supplementary figures and images for: Prognostic value of mesorectal package area in patients with locally advanced rectal cancer following neoadjuvant chemoradiotherapy: A retrospective cohort study
Source: Front Oncol. 2022 Oct 3;12:941786. doi: 10.3389/fonc.2022.941786 (PMC9574388; doi:10.3389/fonc.2022.941786)

Supplementary


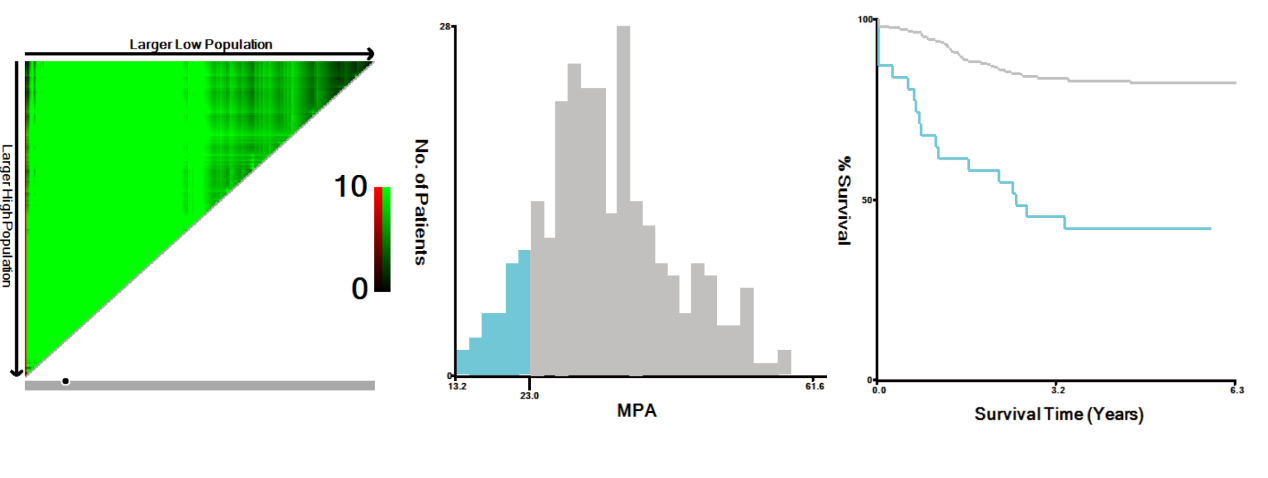


FIGURE 1

MPA cutoff points produced by X-tile plot.

Supplement: Supplementary file 1 [file DataSheet_1.docx]
